# Identification and expression of the WRKY transcription factors of *Carica papaya* in response to abiotic and biotic stresses

**DOI:** 10.1007/s11033-013-2966-8

**Published:** 2014-01-04

**Authors:** Lin-jie Pan, Ling Jiang

**Affiliations:** College of the Department of Horticulture and Forestry of Huazhong Agricultural University, Key Laboratory of Horticultural Plant Biology of Ministry of Education, National Indoor Conservation Center of Virus-free Gemplasms of Fruit Crops, Wuhan, 430070 Hubei China

**Keywords:** *Carica papaya* L., WRKY transcription factor, Quantitative real time PCR (qRT-PCR), Biotic stress, Abiotic stress, Papaya ringspot virus (PRSV)

## Abstract

The WRKY transcription factor (TF) plays a very important role in the response of plants to various abiotic and biotic stresses. A local papaya database was built according to the GenBank expressed sequence tag database using the BioEdit software. Fifty-two coding sequences of *Carica papaya* WRKY TFs were predicted using the tBLASTn tool. The phylogenetic tree of the WRKY proteins was classified. The expression profiles of 13 selected *C. papaya* WRKY TF genes under stress induction were constructed by quantitative real-time polymerase chain reaction. The expression levels of these WRKY genes in response to 3 abiotic and 2 biotic stresses were evaluated. TF_807.3_ and TF_72.14_ are upregulated by low temperature; TF_807.3_, TF_43.76_, TF_12.199_ and TF_12.62_ are involved in the response to drought stress; TF_9.35_, TF_18.51_, TF_72.14_ and TF_12.199_ is involved in response to wound; TF_12.199_, TF_807.3_, TF_21.156_ and TF_18.51_ was induced by PRSV pathogen; TF_72.14_ and TF_43.76_ are upregulated by SA. The regulated expression levels of above eight genes normalized against housekeeping gene *actin* were significant at probability of 0.01 levels. These WRKY TFs could be related to corresponding stress resistance and selected as the candidate genes, especially, the two genes TF_807.3_ and TF_12.199_, which were regulated notably by four stresses respectively. This study may provide useful information and candidate genes for the development of transgenic stress tolerant papaya varieties.

**Electronic supplementary material:**

The online version of this article (doi:10.1007/s11033-013-2966-8) contains supplementary material, which is available to authorized users.

## Introduction

*Carica papaya* is an economically important fruit in southern China as well as other tropic and sub-tropic countries. Its flower bud formation and fruit production are susceptible to abiotic and biotic stresses such as extreme temperatures, seasonal droughts, typhoon wounds, and papaya ringspot virus (PRSV). These stresses may cause severe economic loss in papaya production in China. The development of transgenic Papaya varieties that are more tolerant to these stresses could be an effective approach to the problems.

Plants have multiple mechanisms for adapting to abiotic and biotic stresses in their natural habitats [1, 12]. Research on the responses of plants to their environments has been focused on the gene regulation of transcriptional level. Transcription factors (TFs) are proteins that can activate or restrain the transcription of downstream target genes by binding directly to promoters of target genes in a sequence-specific mode [37]. The WRKY TFs form one of the largest families and play a broad-spectrum regulatory role as positive and negative regulators in the responses to abiotic and biotic stresses in plants [1].

Proteins of the *WRKY* gene family contain one or two highly conserved WRKY domains and a zinc finger motif in the C-terminal region [10]. WRKY proteins containing a single WRKY domain with the C2-H2 (C-X4-5-C-X22-23-H-X1-H) pattern are group I, WRKY proteins containing two WRKY domain followed by a C2H2 are group II; WRKY proteins containing a single WRKY domain with C2-HC (C-X7-C-X23-H-X1-C) pattern are group III; group IV especially for WRKY proteins that contain a WRKY domain but lack a complete zinc finger [10, 41]. The WRKY domain can bind to the TTGAC(C/T) of W-box found in promoters of target genes and regulates its transcription [44]. WRKY family members appear to be involved in the regulation of various physiological and development processes in plants, such as senescence, embryogenesis, regulation of biosynthetic pathways, hormone signaling, etc. [42].

*WRKY* genes are frequently reported to be involved in various stress responses. The WRKY proteins have been observed in response to various pathogenic infections, such as fungal, bacterial, and viral [11, 17, 29, 39]. Some *WRKYs* are induced by pathogen infection, and activated by other elicitors such as SA or wounding [2]. Hwang et al. [15] reported the heterologous expression of *OsWRKY6* gene in Arabidopsis activates the expression of defense related genes and enhances resistance to pathogens. *WRKY* TFs have also been shown to regulate cross-talk between jasmonate- and salicylate-regulated disease response pathways [56]. Different stresses have been reported to induce the expression of various *WRKY* TFs. For example, SA induces *AtWRKY3*, *BnWRKY*, *CaWRKY1*, *FaWRKY1,* *HvWRKY38,* and *OsWRKY* [8, 23, 36, 40, 43, 48, 50]; cold stress induces *HvWRKY* and *LtWRKY* [32, 59, 60]; drought induces *HvWRKY* and *OsWRKY* [40, 43]; and wound induces *LtWRKY*, *OsWRKY*, *OsWRKY*, *PtWRKY*, *VvWRKY*, and *WtWRKY* [24, 31]. Many *WRKY* TFs are activators, such as *AtWRKY3* and *AtWRKY4* [23], *CaWRKYb* [17]. Some *WRKYs*, however, are repressors, such as *AtWRKY62* [22], *OsWRKY51* and *OsWRKY71* [47].

The rapid and effective quantitative real-time polymerase chain reaction (qPCR) is still considered to be the effective method for the comprehensive quantification analysis of WRKY expression at the genome level [21, 52]. Since the identification of the first WRKY protein, SPF1, from sweet potato (*Ipomoea batatas*) [16], large numbers of *WRKY* genes have been cloned from various plant species including potato [6], tobacco [53], wheat and barley [44], *Arabidopsis* [3, 4, 15, 49, 56], pepper [38, 57], grape [31], rice [40, 41, 55], capsicum [36], populus [24], canola [50], Cucumis sativus [27], cotton [52], etc. Although numerous *WRKY* genes have been identified or predicted from many different species, only a small number of them have been functionally characterized in Arabidopsis, soybean, rice, tobacco, etc. [26]. The *WRKY* genes of papaya have been confirmed since the whole genome sequence of the papaya plant has been completed [33].

Dong et al. [7] reported the expression profile of WRKY against pathogenic stress in *Arabidopsis*, and induced expression was detected in 49 out of the 72 tested *WRKY* genes. Ming et al. [33] reported 52 WRKYs in papaya. However, the number of WRKYs in papaya responsible to stresses remains unknown. And the *WRKY* genes have not yet been functionally characterized.

The purpose of present study is to build a local database for *WRKY* genes in papaya, to construct a phylogenetic tree using the domain amino acid sequences of these WRKY proteins, and to detect the expression profiles of selected candidate *WRKYs* under various stressed conditions and predict the possible functions based on their expression patterns. This research may provide useful information and candidate genes for the development of transgenic stress tolerant papaya varieties.

## Materials and methods

### Materials and treatments

Seedlings of *C. papaya* L. ‘Sunup’ provided by the Institute of Agriculture Science in Fujian Province were cultured at 28 °C,under a photoperiod of 14 h/day. Stress treatments were performed on 30-day-old seedlings with four to five leaves. 1 mmol/L SA was sprayed onto the cotyledons and two euphilla at a dose of 10 ml/plant. Afterwards, the leaves were collected 12 h after treatment. Plants treated with only water served as the control. The stress and control groups were kept in different growth chambers. Rubbing quartz sands on the surface of leaves, producing small cuts, performed wound treatment. Keeping the plants at 4 °C for 12 h imposed a low temperature stress, whereas the control plants were grown at 28 °C, at dark. Drought stress was induced by not providing water for 1 week with the quadrate plastic pot of 12 cm in height, and 10 cm in width, whereas the control group was regularly watered. PRSV pathogens were identified by reverse transcriptase (RT)-PCR. The leaves (provided by the Fruit Institute of Guangdong Province) were inoculated with pathogen juice in phosphate-buffered saline (PBS), whereas the control plant was inoculated only with PBS, the samples were collected after 24 h. The leaves were harvested at certain time points as indicated in each experiment, frozen with liquid nitrogen, and stored at −80 °C until RNA extraction. The basal levels of WRKY expression were evaluated and normalized to the *actin* transcript level of papaya. Each treatment involved the leaves of five plants, and samples were taken from experiments conducted in triplicate.

### Database collection and gene annotation

The protein sequence corresponding to each papaya WRKY unigene was determined by SUPERFAMILY (http://supfam.org/SUPERFAMILY/index.html). Utilizing GenBank information, the BLAST local database of the expressed sequence tags (ESTs) of papaya was constructed using the BioEdit software with EST sequences (EX227656–EX303501) for comparison and confirmation of the nucleotide sequence of the *WRKY* genes. The operator procedure is following: download the genomics coding sequence of “Sunup” of papaya from NCBI database, and save the genomics sequences with FASTA file format, a local nucleotide database file was created, it was named “papaya.aa”. To startup BioEdit software program, selected Accessory Application and use BLAST function, and then, paste the amino acid sequence, and selecting blast function and the nucleotide sequence of WRKY ZF were confirmed in papaya. The specific WRKY-type domain signature and WRKYGQK heptapeptide motif were compared using the BioEdit software. The specific ZF WRKY-type domain signatures were also investigated by searching the ExPasy proteomics server (http://cn.expasy.org). The WRKY amino acid sequences were aligned, and a phylogenetic tree was constructed using DNAman software.

### RNA extraction and qRT-PCR analysis

Total RNA was extracted using TRIzol reagent (Invitrogen) following the manufacturer’s instruction, with DNase I digestion for purification. The RNA samples were detected using an Ultrospec 2100 UV/Visible spectrophotometer (Amershan GE Healthcare, USA). First-strand cDNA was synthesized from 2 μg of total RNA in a 20-μL final volume using an M-MLV first-strand cDNA kit. qPCR was performed using Platinum SYBR Green qPCR Super Mix-UDG (Invitrogen) following the manufacturer’s instruction. A real-time qPCR assay for gene expression-specific primers was designed from the papaya cDNA sequences using the Primer Express 5.0 software at 58–60 °C. The amplification fragment lengths were 98–193 bp. The primer sequences are shown in Supplemental Table 2. These primers were designed with Primer 5-cracked software, the primers were synthesized by Sanggon Shanghai Biology Technology, Ltd. qRT-PCR was performed using Rator 6000 (Corbett). The primers were strictly filtrated by reverse transcription test and amplified the single band.

The cycling conditions started with 2 min of polymerase activity at 95 °C and 40 cycles at 95 °C for 20 s, followed by 60 °C for 20 s and 72 °C for 20 s. Each assay was conducted in triplicate, and a no-template control was included. The threshold cycle (Ct) of the primary amplification curve was used for calculations. The *actin* gene was chosen as the internal constitutively expressed control (normalization) according to the formula ∆∆Ct = (C_t, target_ − C_t, *Actin*_) _time *x*_ − (C_t, target_ − C_t, *Actin*_) _time 0_. The relative expression level was analyzed using the 2^−∆∆Ct^ method [30]. Dilutions of cDNA (1:10 to 1:1,000) from a reference sample were used to construct a relative standard curve. The specificity of the PCR products was verified by melting curve analyses (60–95 °C). Only primer sets producing a single sequence-specific peak in the dissociator curve were conserved. The data were analyzed using the Roter Gene 6000 Series software (VIRTUAL Mode software package) to obtain the relative expression levels of the papaya gene based on the comparative Ct method. The significant differences among the data were analyzed via *t* tests using Microsoft Excel. Data are represented as means and standard errors of three replicates.

## Results

### Identification of WRKY TFs and their nucleic acid sequence in papaya

A total of 52 significant WRKY domains in 50 proteins have been predicted using the SUPERFAMILY database of *C. Papaya*. In the present study, after analyzing the homology of the putative amino acid sequence and eliminating redundancies, 52 nucleic acid coding sequences of *C. Papaya* WRKY TFs were identified using the tBLASTn tool. These data were mined from 47483 papaya ESTs in the *C. Papaya* genome. The numbers of TFs, relative GenBank accession numbers, protein size, amino acid positions, frame and available nucleic acid sequence, WRKY type domain signature are shown in Supplement Table 1.

**Fig. 1:**
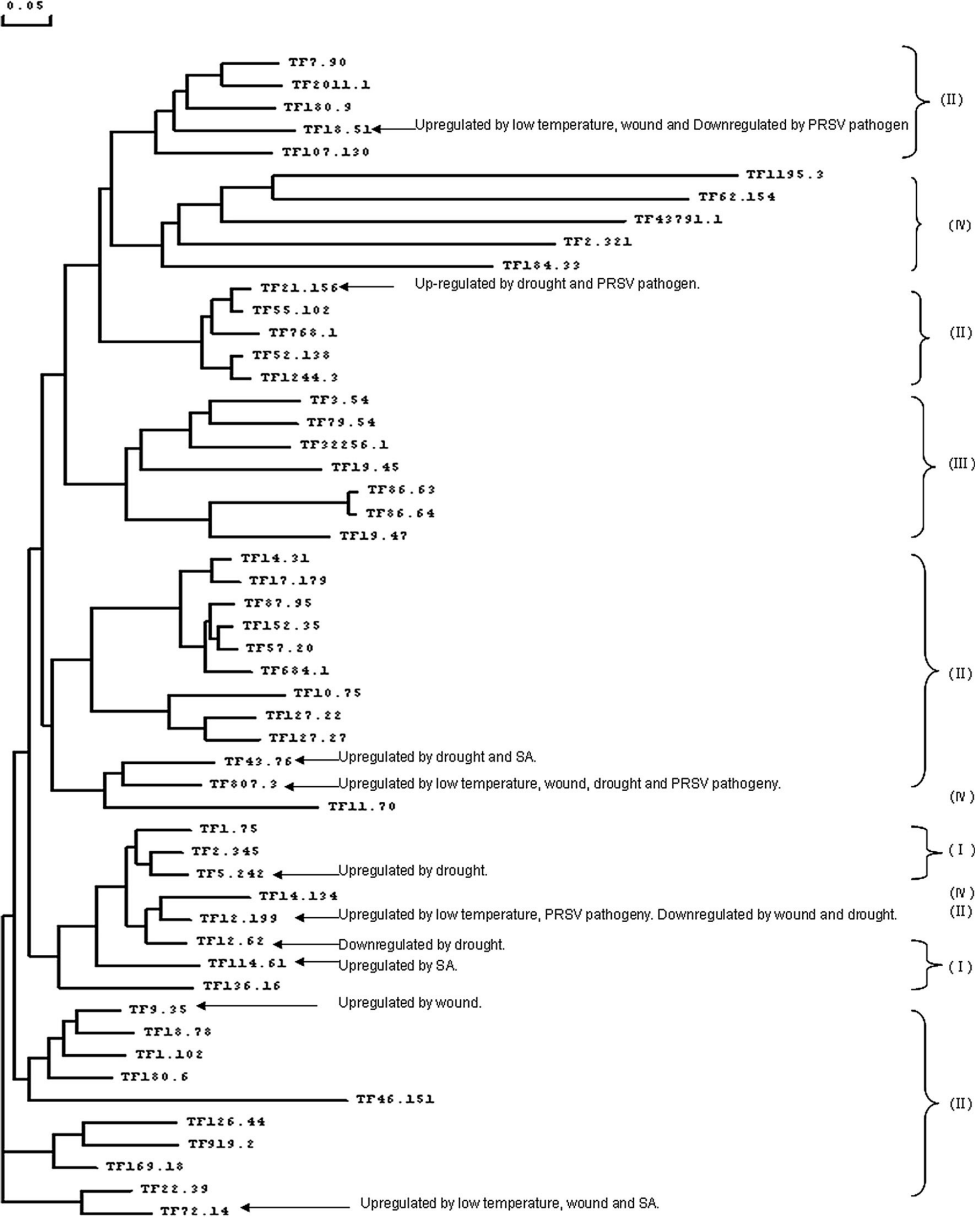
Phylogenetic unrooted tree of the WRKYs in *C. papaya*. Relationships among WRKY TF, as illustrates by phyogenetic tree produced by DNAMAN. WRKYs were classified into groups I, II, III and IV

To examine the evolutionary relationships among the WRKY domains, a phylogenetic tree was constructed using the conserved WRKY domain amino acid sequences. The phylogenetic tree demonstrated that the 52 WRKYs could be classified into 4 groups according to the most prominent feature of these proteins, the WRKY domain, which contained 60 amino acids. Group I includes 6 WRKYs that have two standard WRKYGQK heptapeptide followed by a C2H2. Group II includes 32 WRKYs that have a conserved WRKYGQK heptapeptide followed by a zine finger CX_4–5_CX_22–23_HHX_1_H. Group III includes 7 WRKYs that have a conserved WRKYGQK heptapeptide followed by a C2HC. And group IV includes 5 WRKYs that do not contain the standard WRKYGQK domain and 2 WRKYs that do not contain the zine finger CX_4–5_CX_22–23_HX_1_H. The phylogenetic unrooted tree of the WRKY transcripts was shown in Fig. 1 with notes for induced expressions of 10 WRKYs under abiotic and biotic stresses.

**Fig. 2:**
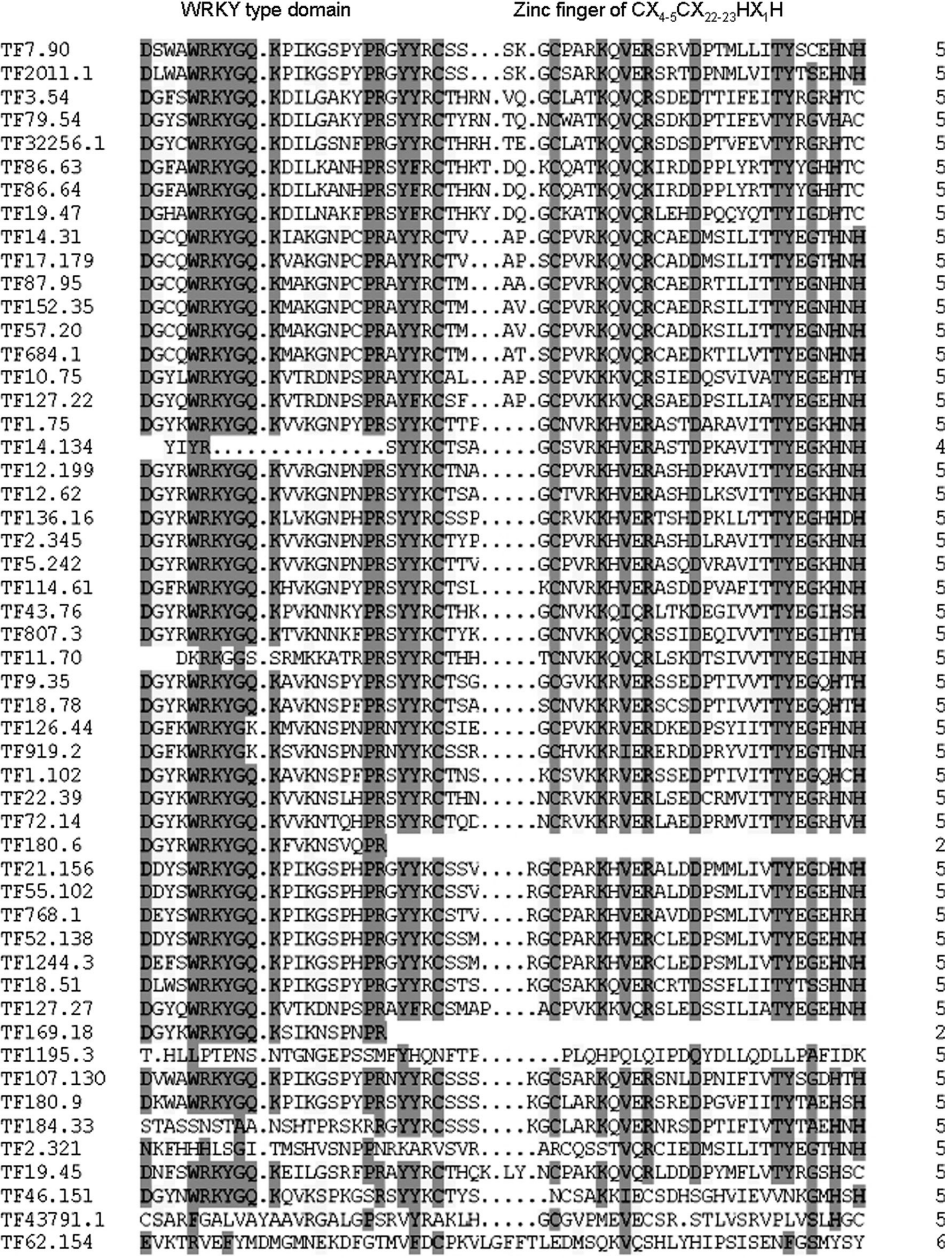
Comparison of WRKY domain and zine figure signature of WRKY domain of papaya

The structural characteristics of the 52 WRKYs was demonstrated by comparing the detailed sequence of 60 amino acids at the N-terminer of the coding sequence containing at least one amino acid motif of WRKY (Fig. 2).

## Expression of *WRKY* genes under abiotic stresses

### Expression of four *WRKYs* is significantly upregulated by low temperature

**Fig. 3:**
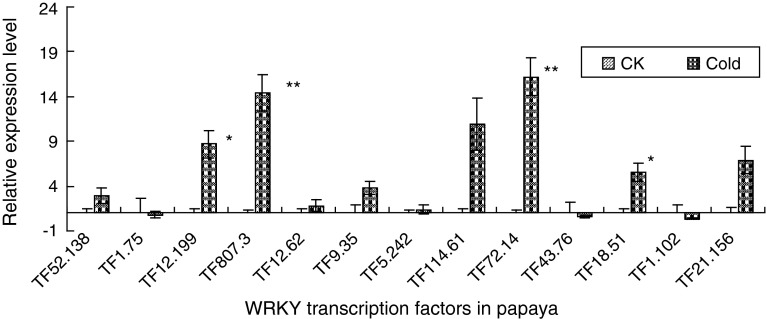
Expression of *WRKY* genes in response to cold stress treatments in papaya. Changes in the WRKY transcript abundance as a result of 4 °C treatment for 12 h

The expression levels of 13 WRKY TFs were analyzed under stress conditions. The results showed that the expression levels of TF_807.3_ and TF_72.14_ were induced by 14.3- and 16.2-fold normalized against housekeeping gene *actin* whose relative mRNA expression was 2^−∆∆Ct^ = 1 (significant at probability <0.01) after 12 h of low-temperature treatment (4 °C). TF_12.199_ and TF_18.51_ were induced by 8.6- and 5.5-fold (significant at probability <0.05) after 12 h of 4 °C treatment. The expression levels of TF_114.61_ and TF_21.156_ were also notably up-regulated, but statistically insignificant (Fig. 3).

### Six WRKYs are involved in the response to drought stress

**Fig. 4:**
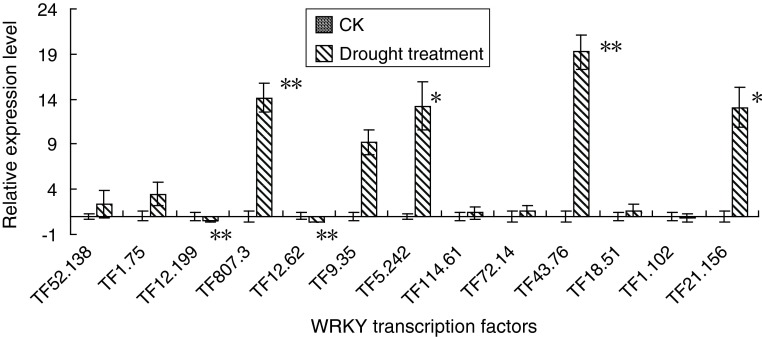
Real-time qPCR of 13 *WRKYs* to analyze their expression following drought treatments

Four *WRKY* genes were upregulated and two WRKYs were down-regulated under drought stress. The expression of TF_807.3_ and TF_43.76_ were increased by 14.12- and 19.22-fold at the significant level of probability <0.01. The expression of TF_5.242_ and TF_21.156_ were increased by 13.2- and 13.1-fold at the significant level of probability <0.05. However, the expression of TF_12.199_ and TF_12.62_ were significantly (*p* < 0.01) decreased by 0.46- and 0.39-fold (Fig. 4).

### Expression of five WRKYs is involved in response to wound

**Fig. 5:**
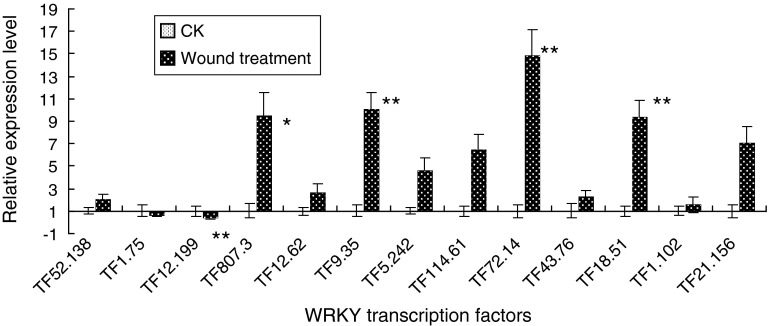
Real-time qPCR of 13 genes to analyze their expression following wound stress treatments for 12 h

Changes in the transcript abundance of the 13 *WRKY* genes in response to wound treatment were examined. The transcript abundances of the *WRKY* genes of TF_9.35_, TF_18.51_ and TF_72.14_ were significantly (*p* < 0.01) increased 12 h after wounding. And the expression of TF_807.3_ were also significantly increased but at a lower probability level (*p* < 0.05). The expression abundance of TF_12.199_, however, was significantly (*p* < 0.05) decreased (Fig. 5).

## Expression of *WRKY* genes under biotic stresses

### Expression of four *WRKY* genes was induced by PRSV pathogen

**Fig. 6:**
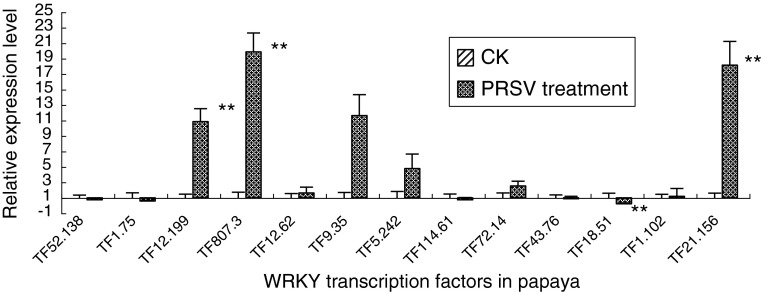
The expression level of *WRKY* after PRSV infection at 24 h

Three *WRKY* genes were upregulated and one *WRKY* were down-regulated when infected by PRSV pathogen. The expression levels of TF_12.199_, TF_807.3_, and TF_21.156_ significantly increased (*p* < 0.01) by 10.8-, 19.8-, and 18.1-fold after 24 h of treatment. The expression level of TF_18.51_ was significantly decreased (*p* < 0.01) by 0.22-fold (Fig. 6).

### Expression of three *WRKY* genes is up-regulated by Salicylic acid

**Fig. 7:**
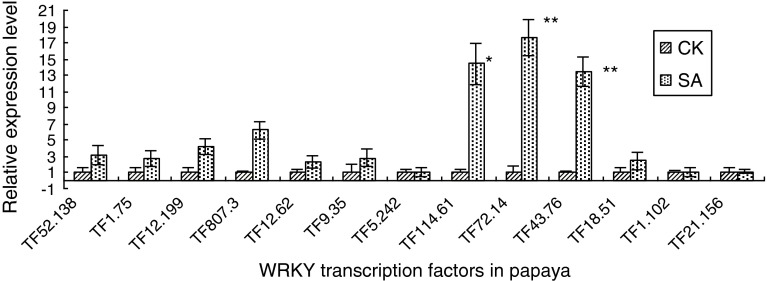
Expression of *WRKYs* in response to SA treatment. Changes in WRKY transcript abundance in response to SA treatment after 12 h

Salicylic acid (SA) plays a critical role in plant defense against pathogens. TF_114.61_, TF_72.14_, and TF_43.76_ were demonstrated to be induced by SA treatment. The transcription abundances of TF_72.14_ and TF_43.76_ were significantly increased (*p* < 0.01) by 17.6- and 13.4-fold at 12 h after the SA treatment, respectively. The transcription abundance of TF_114.61_ was increased by 14.4-fold which is significant at probability <0.05 level (Fig. 7).

### Identification of *WRKYs* in response to abiotic and biotic stresses

A total of ten *WRKYs* were indentified in response to three abiotic and two biotic stresses (Table 1). Four *WRKYs* were up-regulated by low-temperature. Six *WRKYs* responded to drought stress, including four upregulated and two downregulated genes. Five *WRKYs* responded to wound, including four upregulated and one down-regulated genes. Four *WRKYs* were induced by PRSV pathogen, including three up-regulated and one down-regulated genes. And three *WRKYs* were up-regulated by SA.

**Table 1:** Expression of the *WRKYs* in response to abiotic and biotic stresses in papaya

and :The upregulated and down regulated expression levels of *WRKY* genes normalized against housekeeping gene *actin* were significant at probability of 0.01 level

↑ and↓: The upregulated and down regulated expression levels of *WRKY* genes normalized against housekeeping gene *actin* were significant at probability of 0.05 level

A *WRKY* gene may respond to one stress or several different stresses. For example, the expression of TF_807.3_ and TF_12.199_ was found to be in response to four different stresses, respectively. TF_807.3_ was up-regulated by low-temperature, drought, wound and PRSV pathogen. TF_12.199_ was up-regulated by low-temperature and PRSV pathogen but down-regulated by drought and wound. The expression of TF_72.14_ and TF_18.51_ responded to three different stresses, respectively. The expression of TF_21.156_ and TF_43.76_ was up-regulated by two different stresses, respectively. While the expression of TF_12.62_, TF_9.35_, TF_14.61_ and TF_14.61_ were in response to single stress, respectively.

### Homological comparison between WRKYs of papaya and that of other plants

Homological analysis on the detailed sequence of 60 amino acids was made between the ten WRKYs and nine AtWRKY in *Arabidopsis*, seven OsWRKY in rice, seven GmWRKY in soybean, one NtWRKY in tobacco, and one VvWRKY in grape (Supplement Fig. 1). The homology of the WRKY TFs in papaya and the WRKYs with known functions in other plants were analyzed by DNAman. Results indicate that TF_12.199_ shares 100 % homology with GmWRKY3 and GmWRKY6, TF_72.14_ shares 88.5 % homology with AtWRKY33, TF_9.35_ shares 80.3 % homology with OsWRKY3, TF_43.76_ and TF_807.3_ share 78.9 and 75.5 % homology with OsWRKY23 respectively, TF_12.62_ shares 75.4 % homology with OsWRKY53. The high homology suggests that the WRKYs in papaya may have similar functions with their homologous genes in other species.

## Discussion

### Characteristics of the WRKY TFs in *C. papaya*

WRKY TFs contain one or two conserved WRKY domains, which can recognize and bind to the TTGAC(C/T) W-box elements found in the promoters of a large number of plant defense-related genes [9, 20, 51]. The detailed nucleotide sequence information of 52 *WRKY* genes was mined using bio-information methods in this study. This information could be used to facilitate the further research of *WRKY* genes in *C. papaya*.

WRKY TFs can be classified based on both the number of WRKY domains and the features of their zinc-finger motif. WRKY TFs are usually classified into three or four groups. 72 WRKYs have been reported in Arabidopsis, 15 WRKYs belong to group I, 43 belong to group II and 14 belong to group III. 96 WRKYs have been found in rice, 13 WRKYs belong to group I, 45 belong to group II, 32 belong to group III, and 6 belong to group IV [50]. The group II including a conserved WRKYGQK heptapeptide followed by a zine finger CX_4–5_CX_22–23_HHX_1_H is the largest group in most plants [10, 50]. In this research, 52 WRKYs in papaya have been classified into 4 groups, 6 WRKYs belong to group I, 32 WRKYs belong to group II, 7 belong to group III, and 7 belong to group IV. Group II is also the largest in papaya.

Homological analysis between the 10 WRKYs induced by abiotic and biotic stresses and WRKYs with known functions in other plants revealed striking similarities in the conserved sequence of 60 amino acids. TF_12.199_ in papaya has exactly the same sequence of 60 amino acids as that of GmWRKY3 and GmWRKY6 in soybean [58].

In present experiment, 13 WRKY genes with high mRNA abundance were selected as the target for qPCR, 4 WRKYs belong to group I and 9 belong to group II. And 10 out of the 13 WRKYs including 3 WRKYs in group I and 7 WRKYs in group II were found to respond to abiotic and/or biotic stresses. This suggests that the WRKYs of groups I and II in papaya may be more sensitive to stresses.

### Mining the WRKYs relative to the resistance against PRSV pathogen in papaya

A large number of *WRKY* genes are induced by pathogens or plant defense signal molecules. In Arabidopsis, 49 of 72 WRKY genes tested were differentially regulated in plants after infection with an avirulent strain of *P. syringae* or treatment with SA [7]. A few WRKYs were testified to have certain functions in Arabidopsis, and several WRKYs have been proven to possess functions related to disease resistance. For example, NtWRKY3 message was induced rapidly upon infection with TMV in tobacco [2, 51]. AtWRKY70 enhanced the resistance to both *Pseudomonas syringae* and *Erysiphe chichoracearum* [25]. And AtWRKY3, AtWRKY4 and AtWRKY41 had the function of enhancing the resistance to *P. syringae* [14, 23]. In this study, the expression of TF_12.199_, TF_807.3_ and TF_21.156_ were up-regulated and TF_18.51_ was down-regulated trend after PRSV infection. The expression of both TF_43.76_ and TF_72.14_ were up-regulated after SA treatment. 7 out of 13 WRKYs were differentially regulated by PRSV and/or SA in papaya. This suggests the possibility of mining the WRKYs relative to the resistance against PRSV pathogen in papaya.

### Homological and functional comparison between WRKYs in *C. papaya* and other plants

Proteins with similar domains may have the same or similar biologic functions [28]. For example, *NtWRKY3* in tobacco share high homology at the amino acid level with *Arabidopsis AtWRKY4* and *WRKY70*, respectively. *NtWRKY3* was induced rapidly upon infection with TMV [2]. *AtWRKY4* could enhance the resistance to *Pseudomonas syringae* [23]. *AtWRKY70* was induced by SA, JA and could enhance the resistance to *P. syringae* and *Erysiphe chichoracearum* [25]. The three homological genes all have functions in responding to disease resistance.

*AtWRKY33* is a multifunctional TF that is involved in both abiotic and biotic stress responses. *AtWRKY33* regulated the antagonistic relationship between defense pathways mediating responses to *P. syringae* and necrotrophic pathogens [56]. And *AtWRKY33* was up-regulated 14 times after NaCl stress treatment [18]. The sequence of TF_72.14_ shared 88.5 % homology with that of *AtWRKY33*. TF_72.14_ was demonstrated to be induced by low temperature, wound and SA treatment in papaya.

*OsWRKY3* in rice was induced by Botrytis & *P. syringae* infection and SA, JA, ACC. It expressed the resistance to *Pseudomonas syringae* [23]. TF_9.35_ shared 80.3 % homology with *OsWRKY3*. The transcript abundance of TF_9.35_ was significantly increased after wounding.

A number of TFs being activated by abiotic stress could also be induced by pathogen infection [4]. The sequence of TF_12.199_ shared 100 % homology with that of *GmWRKY6* which was related to drought resistance in soybean [58]. The expression level of TF_12.199_ was increased by cold and PRSV, but was decreased by wound and drought treatments. TF_12.199_ could be a multifunctional TF involved in both abiotic and biotic stress responses in papaya.

### The potential application of the WRKYs in *C. papaya*

The WRKY genes in papaya has been studies by analyzing their nucleotide sequence information, classification according to their characteristics of WRKY type domain, and detecting the expression of *WRKY* TFs under three biotic and two abiotic stresses. Ten *WRKYs* have been detected to be in response to the stresses. The regulated expression levels of eight out of ten *WRKYs* are significant at probability of 0.01 levels. These WRKY TFs could be related to corresponding stress tolerance. Two *WRKYs*, TF_807.3_ and TF_12.199_, each regulated by four different stresses, are of especially interesting for further functional verification. This study may provide useful information for the genetic improvement and candidate genes for the development of transgenic stress tolerant papaya varieties.

## Electronic supplementary material

Below is the link to the electronic supplementary material.

Fig. S1 Homology comparing the WRKYs domain among papaya and the following WRKYs in the other plants. WRKYs, accession number, functions and reference showed as following:AtWRKY3 (AT2G38470.1): Resistance to *Pseudomonas syringae* [23]. OSWRKY3 (Loc_Os03g55080): Upregulates pathogenesis-related genes [46]. OsWRKY23 (Loc_Os01g43550): Enhances pathogen defense [20] AtWRKY4 (Loc_Os06g44010): Resistance to *Pseudomonas syringae* [23]. GmWRKY48 (EU019584): Moderate induction of gene expression by drought and cold [58]. OsWRKY13 (Loc_Os09g30400): Resistance to bacterial blight and fungal blast [28]. AtWRKY27 (AAN15550): Influences wilt disease symptom development caused by *Ralstonia solanacearum* [34]. OsWRKY31 (Loc_Os01g53260): Resistance to *Magnaporthe grisea* [55]. AtWRKY45 (AY870611): Increase the resistance against the disease and drought in Arabidopsis [54]. OsWRKY45-1 (AC134346): Enhance resistance to rice blast fungus in rice and weaken *resistance to* rice bacterial blight [54]. AtWRKY70 (AA063359): Resistance to *P*. *syringae* and *Erysiphe chichoracearum* [25]. AtWRKY2 (AAK28313): Seed germination and post germination growth [19], AtWRKY41 (AAN13135): Resistance to Pseudomonas, JA signaling [14]. AtWRKY62 (AAM78067): PstDC3000 inoculation, SA negative regulators of plant basal defense [22]. GmWRKY30 (EU019570): Salt, drought and cold moderate induction of gene expression [58]. OsWRKY71 (DAA05136): Overexpression of *OsWRKY71* in rice resulted in enhanced resistance to virulent *Xoo* 13751. GmWRKY21 (DQ322685): Salt, drought and cold moderate induction of gene expression [58]. AtWRKY25 (AT2G30250):Tolerance to NaCl [20]. VvWRKY2 (AY598466): Wounding, *P*. *viticola* infection regulates [35]. lignifications, xylem development and resistance to fungus [13]. GmWRKY9 (EU019557): Salt and drought moderate induction of gene expression [58]. NtWRKY12 (AAD16139): Induction of PR-1a gene expression by salicylic acid and bacterial elicitors [45]. GmWRKY3 (EU375350): Moderate induced expression to drought stress [58]. GmWRKY6 (DQ322690): Moderate induced expression to drought stress [58]. GmWRKY62 (EU019591): Salt and cold moderate induction of gene expression [58]. OsWRKY53 (AB190436): Induction causing by *Agnaporthe grisea* in *Oryza sativa* L. [5]. Scale represents branch length. “+” represent the WRKY of papaya. (JPEG 127 kb)

Supplementary material 2 (DOC 101 kb)

Supplementary material 3 (DOC 31 kb)

## Abbreviations

AS: Salicylic acid
TF: Transcription factor
TFPs: Transcription factor proteins
ZF: Zinc finger
CDS: Coding sequence
WRKY: Transcriptional regulatory factors in which N-terminal ends contain a conserved WRKYGQR amino acids sequences
PBS: Phosphate buffer solution
