# Supplementary figures and images for: Identification and expression of the WRKY transcription factors of Carica papaya in response to abiotic and biotic stresses
Source: Mol Biol Rep. 2014 Jan 4;41(3):1215–25. doi: 10.1007/s11033-013-2966-8 (PMC3933750; doi:10.1007/s11033-013-2966-8)

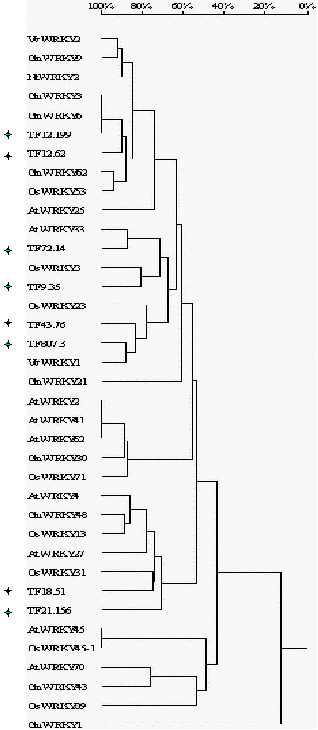

Supplement: Supplementary file 1 — Homology comparing the WRKYs domain among papaya and the following WRKYs in the other plants. WRKYs, accession number, functions and reference showed as following:AtWRKY3 (AT2G38470.1): Resistance to Pseudomonas syringae [23]. OSWRKY3 (Loc_Os03g55080): Upregulates pathogenesis-related genes [46]. OsWRKY23 (Loc_Os01g43550): Enhances pathogen defense [20] AtWRKY4 (Loc_Os06g44010): Resistance to Pseudomonas syringae [23]. GmWRKY48 (EU019584): Moderate induction of gene expression by drought and cold [58]. OsWRKY13 (Loc_Os09g30400): Resistance to bacterial blight and fungal blast [28]. AtWRKY27 (AAN15550): Influences wilt disease symptom development caused by Ralstonia solanacearum [34]. OsWRKY31 (Loc_Os01g53260): Resistance to Magnaporthe grisea [55]. AtWRKY45 (AY870611): Increase the resistance against the disease and drought in Arabidopsis [54]. OsWRKY45-1 (AC134346): Enhance resistance to rice blast fungus in rice and weaken resistance to rice bacterial blight [54]. AtWRKY70 (AA063359): Resistance to P. syringae and Erysiphe chichoracearum [25]. AtWRKY2 (AAK28313): Seed germination and post germination growth [19], AtWRKY41 (AAN13135): Resistance to Pseudomonas, JA signaling [14]. AtWRKY62 (AAM78067): PstDC3000 inoculation, SA negative regulators of plant basal defense [22]. GmWRKY30 (EU019570): Salt, drought and cold moderate induction of gene expression [58]. OsWRKY71 (DAA05136): Overexpression of OsWRKY71 in rice resulted in enhanced resistance to virulent Xoo 13751. GmWRKY21 (DQ322685): Salt, drought and cold moderate induction of gene expression [58]. AtWRKY25 (AT2G30250):Tolerance to NaCl [20]. VvWRKY2 (AY598466): Wounding, P. viticola infection regulates [35]. lignifications, xylem development and resistance to fungus [13]. GmWRKY9 (EU019557): Salt and drought moderate induction of gene expression [58]. NtWRKY12 (AAD16139): Induction of PR-1a gene expression by salicylic acid and bacterial elicitors [45]. GmWRKY3 (EU375350): Moderate induced expression to dro [file 11033_2013_2966_MOESM1_ESM.jpg]
