# Supplementary material for: Identification and expression of the WRKY transcription factors of Carica papaya in response to abiotic and biotic stresses
Source: Mol Biol Rep. 2014 Jan 4;41(3):1215–25. doi: 10.1007/s11033-013-2966-8 (PMC3933750; doi:10.1007/s11033-013-2966-8)
Supplement: Supplementary file 2 — Supplementary material 2 (DOC 101 kb) [file 11033_2013_2966_MOESM2_ESM.doc]

**Supplement Table 1** The available nucleic acid sequence of WRKY transcription factors and Conserved Zinc finger WRKY type domain signature in papaya

| No. | Transcription factors of WRKY | Protein size (aa) | GenBank Accession | Frame and available  Sequence（5'-3'） | Prosite of WRKY in protein (aa) | Zinc finger WRKY type domain signature |
| --- | --- | --- | --- | --- | --- | --- |
| 1 | 12.199 | 422 | gi|186163163|  gi|186163162| | -2, 4085-535  -3, 2180-256 | 233 - 298 | SEVDILDDGYRWRKYGQKVVRGNPNPRSYYKCTNAGCPVRKHVERASHDPKAVITTYEGKHNHDVP |
| 2 | 9.35 | 393 | gi|186164124| | -1, 19609-18033 | 205 - 270 | SEVDHLDDGYRWRKYGQKAVKNSPYPRSYYRCTSGGCGVKKRVERSSEDPTIVVTTYEGQHTHPCP |
| 3 | 57.20 | 490 | gi|186154630| | -3, 23855-23223  -2, 26221-24323 | 239 - 305 | SEAPMISDGCQWRKYGQKMAKGNPCPRAYYRCtMAVGCPVRKQVQRCADDKSILITTYEGNHNHPLP |
| 4 | 5.242* | 582 | gi|186165012| | -2, 18613-18269  -1, 18203-18024  -2, 17665-16229 | 246 – 310  408 - 473 | REQRRAEDGYNWRKYGQKQVKGSENPRSYYKCTFPNCPTKKKVER-SLDGQITEIVYKGSHNHPKP  SDIDILDDGYRWRKYGQKVVKGNPNPRSYYKCTTVGCPVRKHVERASQDVRAVITTYEGKHNHDVP |
| 5 | 2.345* | 609 | gi|186165883| | +2, 1247-2128  +3, 2232-4066 | 254 – 318  432 - 497 | RESRRSDDGYNWRKYGQKQVKGSENPRSYYKCTYPNCPTKKKVER-SLDGQITEIVYKGTHNHPKP  SDIDILDDGYRWRKYGQKVVKGNPNPRSYYKCTYPGCPVRKHVERASHDLRAVITTYEGKHNHDVP |
| 6 | 1.75* | 541 | gi|186166516| | -2, 5631-1621 | 252 – 316  428 - 493 | SIDKPADDGYNWRKYGQKQVKGSEFPRSYYKCTHPSCPVKKKVER-SLDGQITEIIYKGQHNHQPP  SEVDLLDDGYKWRKYGQKVVKGNPYPRSYYKCTTPGCNVRKHVERASTDARAVITTYEGKHNHEVP |
| 7 | 152.35 | 632 | gi|186146379| | -3, 10671-10234  -2, 10162-9377  -3, 8760-8056 | 355 - 421 | SEAPMITDGCQWRKYGQKMAKGNPCPRAYYRCtMAVGCPVRKQVQRCAEDRSILITTYEGNHNHPLP |
| 8 | 114.61* | 464 | gi|186148897| | -3,36680-36453  -1,36382-36254  -2, 36204-35122  -1, 34912-34751 | 187 – 251  385 - 450 | SSDRPSYDGYNWRKYGQKQVKGSEYPRSYYKCTHPNCPVKKKVER-SFDGQIAEIVYKGEHNHPKP  TDSEILDDGFRWRKYGQKHVKGNPYPRSYYRCTSLKCNVRKHVERASDDPVAFITTYEGKHNHDMP |
| 9 | 136.16* | 388 | gi|186147362| | +3, 1101-1697  +2, 1751-1921  +1, 2005-2166  +3, 2403-2669 | 107 – 165  274 - 339 | EDGYNWRKYGQKLVRGNEFIRSYYKCTHPKCLVKKQLER-SHDGRITDTVYFGQHDHPKP  SEVDIVNDGYRWRKYGQKLVKGNPHPRSYYRCSSPGCRVKKHVERTSHDPKLLTTTYEGHHDHDKP |
| 10 | 12.62* | 747 | gi|186163276| | +1, 3106-3555  +2, 4178-4396  +1, 4555-5436  +2, 5522-6388 | 316 – 380  533 - 598 | GGSAPSDDGYNWRKYGQKQVKGSEYPRSYYKCTHPNCQVKKKVER-SHEGHITEIIYKGAHNHPKP  SEVDILDDGYRWRKYGQKVVKGNPNPRSYYKCTSAGCTVRKHVERASHDLKSVITTYEGKHNHDVP |
| 11 | 17.179 | 603 | gi|186161733| | +1, 2383-2454  +2, 3083-3169,  +3, 3585-3725  +2, 4100-4909  +1, 5222-6157 | 250 - 316 | CDTPTMNDGCQWRKYGQKVAKGNPCPRAYYRCtVAPSCPVRKQVQRCADDMSILITTYEGTHNHPLP |
| 12 | 18.78 | 321 | gi|186161663| | +3, 1860-2258  +2, 2396-2566  +1, 2743-3165 | 155 - 220 | SEVDHLEDGYRWRKYGQKAVKNSPFPRSYYRCTSASCNVKKRVERSCSDPTIVVTTYEGQHTHPSP |
| 13 | 52.138 | 349 | gi|186155145| | +2, 2267-3256  +3,4056-4199 | 276 - 342 | KLADIPPDDYSWRKYGQKPIKGSPHPRGYYKCsSMRGCPARKHVERCLEDPSMLIVTYEGEHNHPKL |
| 14 | 807.3 | 186 | gi|186140666| | +2, 3944-4345, 4715-4891 | 102 - 167 | SQIDILDDGYRWRKYGQKTVKNNKFPRSYYKCTYKGCNVKKQVQRSSIDEQIVVTTYEGIHTHSTQ |
| 15 | 87.95 | 618 | gi|186151234| | -3, 27167-26814  -1, 26701-25880  -2, 25785-25669  -3, 25598-24900 | 345 - 411 | SEAPMISDGCQWRKYGQKMAKGNPCPRAYYRCtMAAGCPVRKQVQRCAEDRTILITTYEGNHNHPLP |
| 16 | 22.39 | 192 | gi|186160787| | +3, 4686-5030  +2, 5294-5455, 7136-7297 | 113 - 178 | SDVDVLDDGYKWRKYGQKVVKNSLHPRSYYRCTHNNCRVKKRVERLSEDCRMVITTYEGRHNHTPS |
| 17 | 43.76 | 183 | gi|186156473|  gi|186156472| | +3, 2040-2420  +3, 81-260 | 98 - 163 | SRVDILDDGYRWRKYGQKPVKNNKYPRSYYRCTHKGCNVKKQIQRLTKDEGIVVTTYEGIHSHQIQ |
| 18 | 684.1 | 534 | gi|186141112| | +2, 2069-2335  +3, 2478-2573  +1, 2728-2892  +3, 2988-2892  +2, 4244-5098 | 282 - 348 | SEASMISDGCQWRKYGQKMAKGNPCPRAYYRCtMATSCPVRKQVQRCAEDKTILVTTYEGNHNHPLP |
| 19 | 1244.3 | 357 | gi|186139292| | +3, 4695-5495, 5597-5737  +2, 6827-6973 | 283 - 349 | KVADIPPDEFSWRKYGQKPIKGSPHPRGYYKCsSMRGCPARKHVERCLEDPSMLIVTYEGEHNHSRL |
| 20 | 55.102 | 363 | gi|186154816| | +1, 5350-6417  +3, 6588-6746 | 285 - 351 | KMADIPPDDYSWRKYGQKPIKGSPHPRGYYKCsSVRGCPARKHVERALDDPSMLIVTYEGEHNHSLS |
| 21 | 14.31 | 602 | gi|186162802| | -1, 8552-8391  -2, 8208-8119  -1, 7999-7469  -3, 7364-7248  -2, 7077-6154 | 252 - 318 | CDAPTMNDGCQWRKYGQKIAKGNPCPRAYYRCtVAPGCPVRKQVQRCAEDMSILITTYEGTHNHPLP |
| 22 | 768.1 | 338 | gi|186140812| | -2, 3934-3206  -3, 2523-2395  -2, 2270-2109 | 259 - 325 | KIADIPPDEYSWRKYGQKPIKGSPHPRGYYKCsTVRGCPARKHVERAVDDPSMLIVTYEGEHRHTQP |
| 23 | 126.44 | 179 | gi|186147986| | +2, 1133-1414, 2130-2260  +3, 2922-3044 | 113 - 178 | SEVEILDDGFKWRKYGKKMVKNSPNPRNYYKCSIEGCPVKKRVERDKEDPSYIITTYEGFHNHRTT |
| 24 | 72.14 | 215 | gi|186152807| | -1, 2991-2761, 2343-2095 | 134 - 199 | SDVDVLDDGYKWRKYGQKVVKNTQHPRSYYRCTQDNCRVKKRVERLAEDPRMVITTYEGRHVHSPS |
| 25 | 127.22 | 354 | gi|186147926| | +1 3904-4827  +3 3516-3818 | 191 - 257 | SCLQVVKDGYQWRKYGQKVTRDNPSPRAYFKCsFAPGCPVKKKVQRSAEDPSILIATYEGEHNHPIP |
| 26 | 21.156 | 367 | gi|186160961 | +2, 5075-5920  +1, 6040-6168, 6592-6753 | 297 - 363 | KMADIPPDDYSWRKYGQKPIKGSPHPRGYYKCsSVRGCPARKHVERALDDPMMLIVTYEGDHNHTQP |
| 27 | 1.102 | 313 | gi|186166490| | +2, 18200-18658  +3, 20181-20327  +2, 21254-21622 | 165 - 230 | SEVDHLEDGYRWRKYGQKAVKNSPFPRSYYRCTNSKCSVKKRVERSSEDPTIVITTYEGQHCHHTA |
| 28 | 180.9 | 321 | gi|186145192| | -3, 10691-10308  -2, 10230-10111  -3, 9980-9504 | 142 - 208 | TEDDLTSDKWAWRKYGQKPIKGSPYPRSYYRCsSSKGCLARKQVERSREDPGVFIITYTAEHSHGHP |
| 39 | 2011.1 | 470 | gi|186137401| | -2, 9069-8488  -2, 7566-7423  -1, 6415-5726 | 215 - 281 | SGEVVPSDLWAWRKYGQKPIKGSPYPRGYYRCsSSKGCSARKQVERSRTDPNMLVITYTSEHNHPWP |
| 30 | 107.130 | 391 | gi|186149494| | +1, 6778-8118 | 204 - 270 | TAENLSADVWAWRKYGQKPIKGSPYPRNYYRCsSSKGCSARKQVERSNLDPNIFIVTYSGDHTHPKP |
| 31 | 919.2 | 143 | gi|186140295| | -3，3295-2975，1606-1409 | 52 - 117 | SEMEVMDDGFKWRKYGKKSVKNSPNPRNYYKCSSRGCHVKKRIERERDDPRYVITTYEGTHNHESP |
| 32 | 3.54 | 374 | gi|186165813| | -1， 33738-36427  -3， 36346-36206 | 121 - 189 | GLEGPLDDGFSWRKYGQKDILGAKYPRGYYRCthrNVQGCLATKQVQRSDEDTTIFEITYRGRHTCNPA |
| 33 | 18.51 | 268 | gi|186161682| | +2, 2246-2341  +1, 2494-2643  +1, 2911-3483 | 58 - 118 | SDLWSWRKYGQKPIKGSPYPRGYYRCsTSKGCSAKKQVERCRTDSSFLIITYTSSHNHPGP |
| 34 | 79.54 | 350 | gi|186151982| | +2, 30524-30829  +3, 30909-31787 | 126 - 183 | DDGYSWRKYGQKDILGAKYPRSYYRCtyrNTQNCWATKQVQRSDKDPTIFEVTYRGVH |
| 35 | 7.90 | 261 | gi|186164552| | +1, 33001-33138  +3, 33288-33437  +1, 33577-34080 | 70 - 136 | GETAPPSDSWAWRKYGQKPIKGSPYPRGYYRCsSSKGCPARKQVERSRVDPTMLLITYSCEHNHPWP |
| 36 | 19.47 | 332 | gi|186161464| | -1, 16266-15631  -3, 15508-15068 | 166 - 224 | DDNFSWRKYGQKEILGSRFPRAYYRCthqKLYNCPAKKQVQRLDDDPYMFLVTYRGSHS |
| 37 | 32256.1 | 353 | gi|186135071| | -3， 2593-2270  -2， 2180-2061  -1， 1329-709 | 127 - 189 | EDGYCWRKYGQKDILGSNFPRGYYRCthrHTEGCLATKQVQRSDSDPTVFEVTYRGRHTCNTR |
| 38 | 127.27 | 293 | gi|186147925| | +2, 1634-1936  +2, 2126-2245  +3, 2667-3092 | 116 - 175 | DGYQWRKYGQKVTKDNPSPRAYFRCsMAPACPVKKKVQRSLEDSSILIATYEGEHNHDIH |
| 39 | 86.63 | 261 | gi|186151316| | -3, 18357-18112  -1, 17099-16992  -3, 16908-16474 | 91 - 154 | EAVALVDDGFAWRKYGQKDILKANHPRSYFRCthkTDQKCQATKQVQKIRDDPPLYRTTYYGHH |
| 40 | 19.44 | 286 | gi|186161464| | +1, 13147-13467  +2, 13607-13711  +3, 13944-14402 | 115 - 178 | VVSPLVEDGHAWRKYGQKDILNAKFPRSYFRCthkYDQGCKATKQVQRLEHDPQQYQTTYIGDH |
| 41 | 86.65 | 177 | gi|186151315| | -2, 1843-1211 | 7 - 70 | QTVTLVADGFAWRKYGQKDILKANHPRSYFRCthkNDQKCQATKQVQKIRDDPPLYRTTYYGHH |
| 42 | 10.75 | 306 | gi|186163787| | +1, 1576-1671  +3, 1809-1856  +3, 2142-2636  +3, 3288-3635 | 149 - 215 | DTSLIVKDGYLWRKYGQKVTRDNPSPRAYYKCaLAPSCPVKKKVQRSIEDQSVIVATYEGEHTHPME |
| 43 | 46.151 | 329 | gi|186155970| | +2, 9710-9922  +1, 10012-10062, 13222-13950 | 183 - 241 | SDGYNWRKYGQKQVKSPKGSRSYYKCTYSNCSAKK-IECSDHSGHVIEVVNKGMHSHDPP |
| 44 | 11.70 | 160 | gi|186163540| | -3, 365-60  -2, 17720-175064 | 88 - 141 | RKGGSSRMKKATRPRSYYRCTHHTCNVKKQVQRLSKDTSIVVTTYEGIHNHPCE |
| 45 | 14.134 | 97 | gi|186162690| | -2, 3897-3601 | 4 - 43 | RSYYKCTSAGCSVRKHVERASTDPKAVITTYEGKHNHDVP |
| 46 | 184.33 | 298 | gi|186145010|  gi|186145011| | +1, 43-558  +1, 6976-7396 | 127 - 167 | RGYYRCsSSKGCLARKQVERNRSDPTIFIVTYTAEHNHPAP |
| 47 | 180.6 | 115 | gi|186145194| | -3,11409-11065 | 89 - 115 | SADDILDDGYRWRKYGQKFVKNSVQPR |
| 48 | 169.18 | 126 | gi|186145607| | +2, 2528-3025 | 100 - 126 | CGNGMADDGYKWRKYGQKSIKNSPNPR |
| 49 | 1195.3 | 118 | gi|186139447| | -2, 3149-2796 | 1 - 29 | MVKKRVERSFQDPSTVITTYEGQHNHQCP |
| 50 | 2.321 | 375 | gi|186166134|  gi|186166133| | +3,34299-34478, 34725-35411  +3, 33-488 | 233 - 256 | VQRCIEDMSILITTYEGTHNHPLP |
| 51 | 43791.1 | 182 | gi|186139374| | -1, 2060-1881  -3,1944-1807 |  | No WRKY type domain |
| 52 | 62.154 | 118 | gi|186139447| | -2, 3149-2796 |  | No WRKY type domain |

*: Contained two zinc fingers WRKY type domain signature
