# Supplementary material for: Identification and expression of the WRKY transcription factors of Carica papaya in response to abiotic and biotic stresses
Source: Mol Biol Rep. 2014 Jan 4;41(3):1215–25. doi: 10.1007/s11033-013-2966-8 (PMC3933750; doi:10.1007/s11033-013-2966-8)
Supplement: Supplementary file 3 — Supplementary material 3 (DOC 31 kb) [file 11033_2013_2966_MOESM3_ESM.doc]

**Supplemental Table 2**

**Supplemental Table 2 The nucleotide sequence of** primers used for qRT-PCR

| **Primer number** | **Nucleotide sequence(5’to 3’)** | **Product size** | **Location in the Genebank** |
| --- | --- | --- | --- |
| 1.102+ | tccgagcgggtcttgttct | 157 | gi|186163625 |
| 1.102- | tcccttcttcctgactttattcg |  |  |
| 1.75+ | cgaataagcgagcaaaggataa | 159 | gi|186166516 |
| 1.75- | gttcaggtgtaggatgggtcaa |  |  |
| 114.61+ | gatgaacccagaagcaaaagaa | 117 | gi|186148897 |
| 114.61- | aatctcagaatcagtggaaccgt |  |  |
| 12.199+ | catacgaggggaagcataatcat | 177 | gi|186163163 |
| 12.199- | gctgctgctcgctcaatct |  |  |
| 12.62+ | ggttcatccgtatttagcacagc | 158 | gi|186163276 |
| 12.62- | gtccaagcggcagcctact |  |  |
| 18.51+ | atgcagcacatcgaagggtt | 98 | gi|186161682 |
| 18.51- | ggttatggctggaagtgtaggtta |  |  |
| 21.156+ | cttctagtcgatgtcattgctcc | 195 | gi|186160961 |
| 21.156- | caggacaccctctgacgcta |  |  |
| 43.76+ | gatgatcggatcaagatccatg | 137 | gi|186156473 |
| 43.76- | cgacttcttgtctgaaacgcata |  |  |
| 5.242+ | ttctcgttggggaagactgg | 130 | gi|186165012 |
| 5.242- | atggttgactattgtattggctga |  |  |
| 52.138+ | gtttgggaagcccttcttttg | 123 | gi|186155145 |
| 52.138- | gcctttgttggagctgtttatg |  |  |
| 72.14+ | ggttaggcagggattattagagg | 115 | gi|186152807 |
| 72.14- | tggtaaagtgaaggaggtgttga |  |  |
| 807.3+ | tcgatggaggaaatatgggca | 116 | gi|186140666 |
| 807.3- | caatggagctgcgttggact |  |  |
| 9.35+ | cagtggaggatgcggagtg | 146 | gi|186164124 |
| 9.35- | ttggattctgagggaggattg |  |  |
| actin+ | ttgattttgagcaggagcttga | 193 | gi|186145364 |
| actin- | tgagtgatggctggaagagaac |  |  |

Note:”+” express the forward primer, the “-” express the reverse primer
